# Neutrophil Extracellular Traps in ST-Segment Elevation Myocardial Infarction: Reduced by Tocilizumab and Associated With Infarct Size

**DOI:** 10.1016/j.jacadv.2024.101193

**Published:** 2024-08-15

**Authors:** Kristine Mørk Kindberg, Kaspar Broch, Geir Øystein Andersen, Anne Kristine Anstensrud, Sissel Åkra, Sindre Woxholt, Ingvild Maria Tøllefsen, Thor Ueland, Brage Høyem Amundsen, Nils-Einar Kløw, Bente Halvorsen, Tuva B. Dahl, Camilla Huse, Sarah Louise Murphy, Jan Kristian Damås, Anders Opdahl, Rune Wiseth, Lars Gullestad, Pål Aukrust, Carlos Santos-Gallego, Ingebjørg Seljeflot, Mathis Korseberg Stokke, Ragnhild Helseth

**Affiliations:** a Department of Cardiology, Center for Clinical Heart Research, Oslo University Hospital Ullevaal, Oslo, Norway; b Faculty of Medicine, Institute of Clinical Medicine, University of Oslo, Oslo, Norway; c Department of Cardiology, Oslo University Hospital Rikshospitalet, Oslo, Norway; d K. G. Jebsen Cardiac Research Centre, University of Oslo, Oslo, Norway; e Department of Cardiology, Oslo University Hospital Ullevaal, Oslo, Norway; f Clinic of Cardiology, St. Olav’s Hospital, Trondheim University Hospital, Trondheim, Norway; g Department of Circulation and Medical Imaging, Norwegian University of Science and Technology (NTNU), Trondheim, Norway; h Research Institute of Internal Medicine, Oslo University Hospital Rikshospitalet, Oslo, Norway; i K. G. Jebsen Thrombosis Research and Expertise Center (TREC), The Arctic University of Norway, Tromsø, Norway; j Department of Radiology, Oslo University Hospital Ullevaal, Oslo, Norway; k Cardiovascular Division, Department of Medicine, Brigham and Women’s Hospital, Harvard Medical School, Boston, Massachusetts, USA; l Department of Infectious Disease, St. Olavs Hospital, Trondheim University Hospital, Trondheim, Norway; m Department of Clinical and Molecular Medicine, Centre of Molecular Inflammation Research, Norwegian University of Science and Technology (NTNU), Trondheim, Norway; n AtheroThrombosis Research Unit, Cardiovascular Institute, Icahn School of Medicine at Mount Sinai, New York, New York, USA; o Institute for Experimental Medical Research, Oslo University Hospital and University of Oslo, Oslo, Norway

**Keywords:** acute myocardial infarction, ischemia/reperfusion injury, inflammation, IL-6, NETs

## Abstract

**Background:**

Interleukin-6-receptor inhibition with tocilizumab improves myocardial salvage in patients with ST-segment elevation myocardial infarction (STEMI). Reduced levels of neutrophil extracellular traps (NETs), which consist of nuclear material studded with proteins released upon neutrophil activation, might contribute to this effect.

**Objectives:**

The purpose of this study was to evaluate the effect of tocilizumab on NETs and investigate the association between NETs and myocardial injury in patients with STEMI.

**Methods:**

In the ASSAIL-MI study, 199 patients with STEMI were randomized to tocilizumab or placebo during percutaneous coronary intervention. In this substudy, we analyzed blood levels of the NET markers double-stranded deoxyribonucleic acid (dsDNA), myeloperoxidase-DNA, and citrullinated histone 3 (H3Cit) at admission and after 24 hours and 3 to 7 days. In a subgroup of patients, we assessed regulation of transcripts related to the formation of NETs. We also investigated associations between NET markers and the myocardial salvage index (MSI).

**Results:**

All NET markers were lower in the tocilizumab group than in the placebo group at 3 to 7 days (all *P* < 0.04). Several NET-related pathways were downregulated in the tocilizumab group. The beneficial effect of tocilizumab on the MSI seemed to be partly dependent on reduction of NETs (structural equation modeling: 0.05, *P* = 0.001 [dsDNA] and 0.02, *P* = 0.055 [H3Cit]). Patients with NETs in the 3 lowest quartiles had higher MSI than patients in quartile 4 (10.9 [95% CI: 4.0-15.0] [dsDNA] and 8.9 [95% CI: 2.0-15.9] [H3Cit], both *P* = 0.01).

**Conclusions:**

NETs were reduced by tocilizumab and associated with myocardial injury. The effect of tocilizumab on MSI might be mediated through reduced NETs. (ASSessing the Effect of Anti-IL-6 Treatment in Myocardial Infarction: The ASSAIL-MI Trial [ASSAIL-MI]; NCT03004703)

Ischemia reperfusion injury is responsible for up to 50% of the final infarct size after ST-segment elevation myocardial infarction (STEMI).^1^ The pathophysiological mechanism involves activation of innate immunity, but treatment options remain elusive.^2^ In the ASSessing the effect of Anti-IL-6 treatment in Myocardial Infarction (ASSAIL-MI) trial, we showed that a single dose of the interleukin (IL)-6 receptor inhibitor tocilizumab attenuated myocardial injury in patients with acute STEMI. Tocilizumab infusion started prior to and continued alongside primary percutaneous coronary intervention with an infusion time of 1 hour. Tocilizumab significantly increased myocardial salvage index (MSI),^3^ ie, the fraction of the ischemic myocardium (area at risk) rescued from necrosis.^4^ There was also a numerically, although not statistically significant, difference in infarct size quantified by cardiac magnetic resonance imaging and peak levels of troponin T between the treatment groups. Subsequent analyses showed that treatment with tocilizumab was associated with a fall in neutrophil cell count as well as attenuated inflammatory potential of the residual neutrophils, including degranulation.^5^ Neutrophils produce neutrophil extracellular traps (NETs),^6^ which are filamentous, thread-like structures of double-stranded deoxyribonucleic acid (dsDNA) twined around histones like citrullinated histone 3 (H3Cit). NETs are embedded with granule proteins including myeloperoxidase (MPO-DNA complexes), and are suggested to contribute to the myocardial injury during ischemia/reperfusion by promoting microvascular obstruction as well as myocardial inflammation and cytotoxicity.^7^, ^8^, ^9^, ^10^ These myocardial NET effects are supplementary to the well-known prothrombotic effects of NETs in atherosclerotic plaques and intracoronary thrombosis that involve interaction between neutrophils, platelets, and endothelial cells.^11^, ^12^, ^13^ Interestingly, targeting NETs with DNAse 1 has been shown to accelerate ex vivo tissue plasminogen activator-induced thrombolysis.^14^

We have previously shown that level of the NET marker dsDNA was associated with infarct size and death in patients with STEMI.^15^^,^^16^ Our aim in the present study was to explore how tocilizumab influenced circulating NET levels in the setting of ischemia reperfusion injury in STEMI patients. Because the IL-6 pathway is thought to stimulate NETosis,^17^ we hypothesized that treatment with the IL-6 receptor antagonist tocilizumab would be associated with reduced circulating levels of NET markers, again contributing to myocardial salvage.

## Methods

### Ethical considerations

The trial protocol is approved by the regional ethics committee (REK Sør-Øst 2016/1223), and all participants provided written informed consent. The safety of the trial was monitored by an independent Data and Safety Monitoring Board. The trial adhered to the principles of the Declaration of Helsinki and followed the guidelines for good clinical practice. Prior to enrolling participants, the trial was registered at ClinicalTrials.gov, NCT03004703.

### Study design

We analyzed data from patients enrolled in the ASSAIL-MI trial between March 16, 2017, and February 13, 2020. The design of ASSAIL-MI has been published previously.^3^ The trial was conducted at 3 high-volume percutaneous coronary intervention centers in Norway and designed as a randomized, double-blind, placebo-controlled trial. The primary objective was to test the effect of a single dose of the IL-6 receptor inhibitor tocilizumab, administered at admission and continued during primary percutaneous coronary intervention, on myocardial injury after STEMI. Patients aged 18 to 80 years with a first-time STEMI and symptoms <6 hours were included. Exclusion criteria were previous myocardial infarction, cardiogenic shock, resuscitated cardiac arrest, left bundle branch block, renal or liver failure, current or chronic infection, current or chronic autoimmune or inflammatory diseases, recent major surgery, or immunosuppressive treatment other than a low dose corticosteroids equivalent to 5 mg prednisolone daily. Patients were randomized 1:1 to a single dose of 280 mg tocilizumab or 1,000 ml of NaCl as placebo, administered as an intravenous infusion over 1 hour starting prior to the percutaneous coronary intervention. The primary endpoint was the MSI as assessed by cardiac magnetic resonance imaging 3 to 7 days after randomization.

### Blood sampling protocol

Blood samples were collected at admission (baseline), after 24 hours, 3 to 7 days, and at 3 and 6 months. Peripheral venous blood was drawn into tubes containing ethylenediaminetetraacetic acid for plasma analysis and without any additives for serum analysis. The ethylenediaminetetraacetic acid tubes were promptly placed on ice and centrifuged within 30 minutes at 2,500 g for 20 minutes at 4 °C to obtain platelet-poor plasma. Tubes without additives were left at room temperature for 30 to 60 minutes to ensure full coagulation before being centrifuged at 2,100 g for 10 minutes at room temperature. Immediately after centrifugation, plasma and serum were aliquoted and stored at −80 °C pending analyses. Filled BD PAXgene Blood RNA tubes were placed at room temperature for 2 to 72 hours before being stored at −80 °C.

### Laboratory analyses

High-sensitivity troponin T was measured 4 times over the first 24 hours and again at 3 to 7 days. We quantified Troponin T by electrochemiluminescence immunoassay (Elecsys 2010 analyzer, Roche Diagnostics). Peak Troponin T was the highest registered value. dsDNA was measured in ethylenediaminetetraacetic acid plasma using a fluorescent nucleic acid stain, Quant-iT PicoGreen (Invitrogen), and quantified through fluorometry (Fluoroskan Ascent, Thermo Fisher Scientific Oy). MPO-DNA complexes were assessed in undiluted serum using an ethylenediaminetetraacetic acid technique previously described by Kessenbrock et al.^18^ Briefly, plates were coated with the capture antibody anti-MPO (Bio-Rad Hercules) and incubated overnight at 4 °C. After blocking with bovine serum albumin, patient samples and a peroxidase-labeled anti-DNA antibody (Cell Death Detection Kit, Roche Diagnostics GmbH) were added and incubated for 2 hours. Subsequently, a peroxidase substrate was added, and absorbance was measured and expressed as optical density (OD) units. H3Cit was analyzed in serum in dilution 1:2 in ethylenediaminetetraacetic acid buffer. A commercial sandwich ethylenediaminetetraacetic acid kit (Cayman Chemical) was used, and performed according to the manufacturer. Interassay coefficients of variation (CVs) were 4.8% (dsDNA), 5.3% (MPO-DNA), and 11.9% (H3Cit). See Supplemental Table 1 for technical specifications on the MPO-DNA and H3Cit enzyme-linked immunosorbent assay.

Total RNA was isolated from BD PAXgene Blood RNA tubes (BD Biosciences) using MagMAX for Stabilized Blood Tubes RNA Isolation Kit (Invitrogen) following the manufacturer's instructions. RNA isolation, RNA sequencing, and bioinformatic analysis were performed as previously described in detail by Huse et al.^5^ Neutrophil-imputed genes ^5^ were imported into Rstudio (v.2022.12.0+353). We performed an over-representation analysis on the “Neutrophil Extracellular Trap Formation pathway” (hsa04613) from the Kyoto Encyclopaedia of Genes and Genomes (KEGG). The analysis involved preranking of genes based on their log2fold difference between the tocilizumab and placebo group. We utilized the cluster Profiler (v.4.6.2) package and performed the analysis with the enrichKEGG function, considering results with an adjusted *P* value <0.05 as statistically significant. Visualization of pathway over-representation analysis results were presented with the *pathview* function. Visual presentation of differentially regulated neutrophil-imputed genes, determined by a t-test *P* value <0.05, belonging to the NETs formation pathway was procured with the ComplexHeatmap (v.2.14.0) package.

### Cardiac magnetic resonance imaging

Patients were examined with cardiac magnetic resonance imaging at 3 to 7 days and after 6 months, performed on 1.5-T systems (Siemens Avanto, Philips Ingenia). With gadolinium contrast, the short-axis images of the left ventricle were obtained both 5 minutes and 15 minutes after contrast administration. All images were analyzed at the Department of Circulation and Medical Imaging, Norwegian University of Science and Technology, Trondheim, Norway, using the Segment software (Medviso). Left ventricular ejection fraction, mass, and volume were analyzed according to recommendations. Infarct size was measured using the expectation maximization, weight intensity, a priori information method with manual correction, and is stated as a percentage of the left ventricle. The area at risk was quantified using short-axis early contrast-enhanced images.^19^ MSI (in %) was calculated as the difference between area at risk and infarct size, divided by area at risk × 100. Microvascular obstruction was defined as areas with absence of gadolinium enhancement within the infarct zone. The area was manually traced and reported as percentage of the left ventricular mass.

### Statistics

The demographic data are given as median (25%, 75% percentiles), mean ± SD, or numbers (%) as appropriate. Wilcoxon sum-rank test, 2 sample t-test, and chi-square test were used to analyze differences between groups. Differences across all time points were assessed by Friedman’s test, followed by the Wilcoxon signed rank test for differences between 2 time points. Correlation analyses were performed by Spearman’s Rho. Outliers have been removed in some figures to optimize the visual presentation, but they are included in the statistical analyses. Supplemental Figure 1 shows the figures with outliers. The direct and indirect effects of tocilizumab were estimated with a structural equation modeling approach using the maximum likelihood optimization algorithm. Linear and logistic regression models were used to investigate associations with the MSI, infarct size, and microvascular obstruction. We adjusted the models for the covariates age, sex, and tocilizumab treatment. We chose not to adjust for inflammatory parameters or troponin, as these are closely linked to NETs and infarct size. The level of statistical significance was set to 2-sided *P* < 0.05. All statistical analyses were performed on STATA v.17 SE (StataCorp LLC), except for RNA sequencing data analysis, which was performed on R version 4.2.1.

## Results

### Study population

A total of 199 patients were enrolled in ASSAIL-MI. The majority were men (85%), and the mean age was 61 years. One in 3 had no known prior disease, and only 6% had established cardiovascular disease (Table 1). Baseline characteristics were equally distributed between the treatment groups. No patients died or developed clinical heart failure during the 6-month follow-up.^3^ One hundred and seventy-eight patients (89%) had cardiac magnetic resonance imaging performed at days 3 to 7 and blood samples eligible for analyzing NET markers (tocilizumab n = 91 and placebo n = 87).

**Table 1:** Baseline Characteristics

	All (N = 199)	MRI and NET Markers Available (N = 178)	
		Tocilizumab (n = 91)	Placebo (n = 87)
Demographics			
Age, y	61 ± 9	62 ± 10	60 ± 9
Male	167 (84%)	72 (79%)	76 (87%)
Body mass index, kg/m^2^	27 ± 4	27 ± 5	28 ± 4
Caucasian	193 (97.0%)	89 (95%)	83 (95%)
Smoking			
Never	74 (37.2%)	35 (38%)	31 (36%)
Current	68 (34.2%)	28 (31%)	35 (40%)
Previous	57 (28.6%)	28 (31%)	21 (24%)
Prior conditions			
Angina pectoris	2 (1%)	1 (1%)	1 (1%)
Cerebrovascular disease	6 (3%)	3 (3%)	2 (2%)
Diabetes mellitus	14 (7%)	7 (8%)	6 (7%)
Hypertension	65 (32%)	30 (33%)	29 (33%)
Treatment			
ACE inhibitor or ARB	47 (24%)	20 (22%)	24 (28%)
Aldosterone antagonist	1 (0.5%)	0 (0%)	1 (1%)
Oral anticoagulation	7 (4%)	4 (4%)	2 (2%)
Platelet inhibitor	17 (9%)	10 (11%)	4 (5%)
Beta blocker	11 (6%)	7 (8%)	3 (3%)
Calcium antagonist	23 (12%)	12 (13%)	10 (11%)
Diuretic	16 (8%)	7 (8%)	8 (9%)
Statin	28 (14%)	17 (19%)	8 (9%)
Clinical characteristics			
SBP at admission, mm Hg	131 ± 23	131 ± 22	131 ± 22
DBP at admission, mm Hg	82 ± 17	81 ± 16	84 ± 16
Heart rate at admission, beats/min	72 ± 16	72 ± 18	70 ± 15
Time from symptom onset to arrival at PCI center, min	135 (95, 185)	135 (95, 185)	135 (100, 185)
Killip class			
I	191 (96%)	85 (93%)	86 (99%)
II-IV	8 (4%)	3 (3%)	4 (5%)
Culprit coronary artery			
Left anterior descending branch	74 (37%)	34 (37%)	29 (33%)
Circumflex or marginal	24 (12%)	11 (12%)	12 (14%)
Right coronary artery	93 (49%)	42 (48%)	43 (51%)
Other	8 (4%)	4 (4%)	3 (3%)
Laboratory values			
White blood count 10^9^/l, admission	11.6 ± 3.4	11.4 ± 3.6	11.6 ± 3.3
Neutrophil granulocytes 10^9^/l, admission	8.6 ± 3.3	8.5 ± 3.4	8.5 ± 3.3
Troponin T ng/l, admission	49 (23, 136)	53 (22, 185)	49 (23, 95)
Peak troponin T ng/l	3,921 (1,637, 7,491)	2,936 (1,432, 6,789)	4,496 (1,937, 8,237)
NT-proBNP ng/l, admission	74 (50, 169)	83 (50, 212)	63 (50, 155)
Creatinine ng/l, admission	76 ± 19	74 ± 17	79 ± 21
HbA1c mmol/mol, admission	37 (34, 40)	37.4 (34, 41)	36.6 (34, 40)
LDL cholesterol mmol/l, admission	3.7 ± 1.0	3.7 ± 1.1	3.7 ± 0.9
C-reactive protein mg/l, admission	2.7 (1.2, 5.0)	2.2 (0.9, 5.0)	2.8 (1.4, 5.0)

Values are mean ± SD, n (%), or median (25th, 75th percentiles).

ACE = angiotensin-converting enzyme; ARB = angiotensin receptor blocker; DBP = diastolic blood pressure; HbA1c = glycated hemoglobin A1c; LDL = low-density lipoprotein; MRI = magnetic resonance imaging; NET = neutrophil extracellular trap; NT-proBNP = N-terminal pro-B-type natriuretic peptide; PCI = percutaneous coronary intervention; SBP = systolic blood pressure.

### Tocilizumab treatment and NETs: serum markers

**Figure 1:**
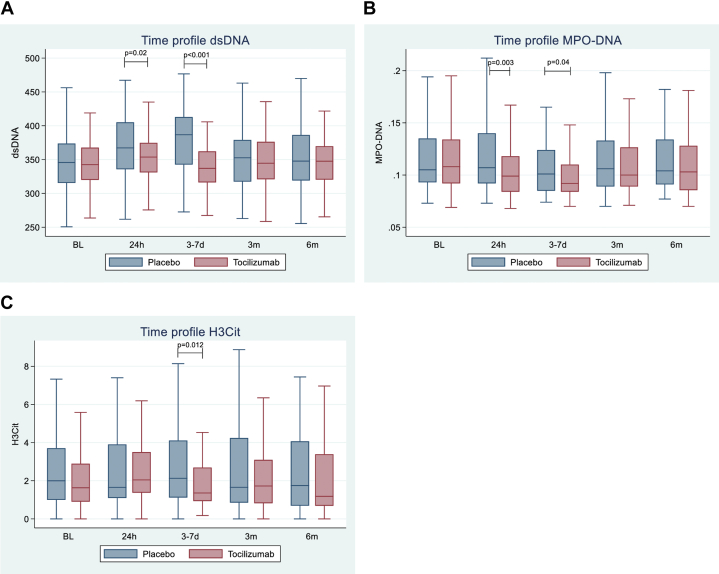
**Time Profiles of NET Markers by Treatment Arms** Levels of (A) dsDNA, (B) MPO-DNA, and (C) H3Cit at baseline, 24 hours, 3 to 7 days, and 3 and 6 months. Median levels, 25- and 75-percentiles, and total range. *P* values of the Wilcoxon rank-sum test, comparing tocilizumab vs placebo. dsDNA = double-stranded deoxyribonucleic acid; H3Cit = citrullinated histone 3; MPO-DNA = myeloperoxidase-deoxyribonucleic acid; NET = neutrophil extracellular trap.

Serum levels of dsDNA, MPO-DNA, and H3Cit levels from baseline to 6 months in the 2 randomized groups are shown in Figure 1. Levels of all markers were similar between the 2 treatment groups at baseline. However, levels of dsDNA and MPO-DNA were lower in the tocilizumab group than in the placebo group at 24 hours and 3 to 7 days (dsDNA: 354 vs 367 ng/ml [24 hours] and 337 vs 387 ng/ml [3-7 days]; MPO-DNA: 0.099 vs 0.107 OD [24 hours] and 0.092 vs 0.101 OD [3-7 days], all *P* ≤ 0.04) (Figure 1). While unaffected at 24 hours, H3Cit levels were lower in the tocilizumab than in the placebo group at 3 to 7 days (1.36 vs 2.13 ng/ml, *P* = 0.012). The change from baseline to 3 to 7 days differed between the groups for dsDNA levels (tocilizumab −7 ng/ml vs placebo 35 ng/ml, *P* < 0.001), but not for MPO-DNA and H3Cit. After 3 and 6 months, there were no differences between the treatment groups for any of the markers.

### NET markers: intercorrelation and association with neutrophil counts and platelets

dsDNA and MPO-DNA intercorrelated weakly at baseline and 24 h (r = 0.162 and 0.214, both *P* < 0.03). MPO-DNA and H3Cit correlated only at baseline (r = 0.224, *P* < 0.002), while dsDNA and H3Cit correlated only after 3 to 7 days (r = 0.187, *P* = 0.001). All NET markers were moderately positively correlated to corresponding neutrophil cell count, and there was no correlation to platelet count (Supplemental Table 2).

### Tocilizumab treatment and NETs: modulation of NET-related pathways in neutrophils

**Figure 2:**
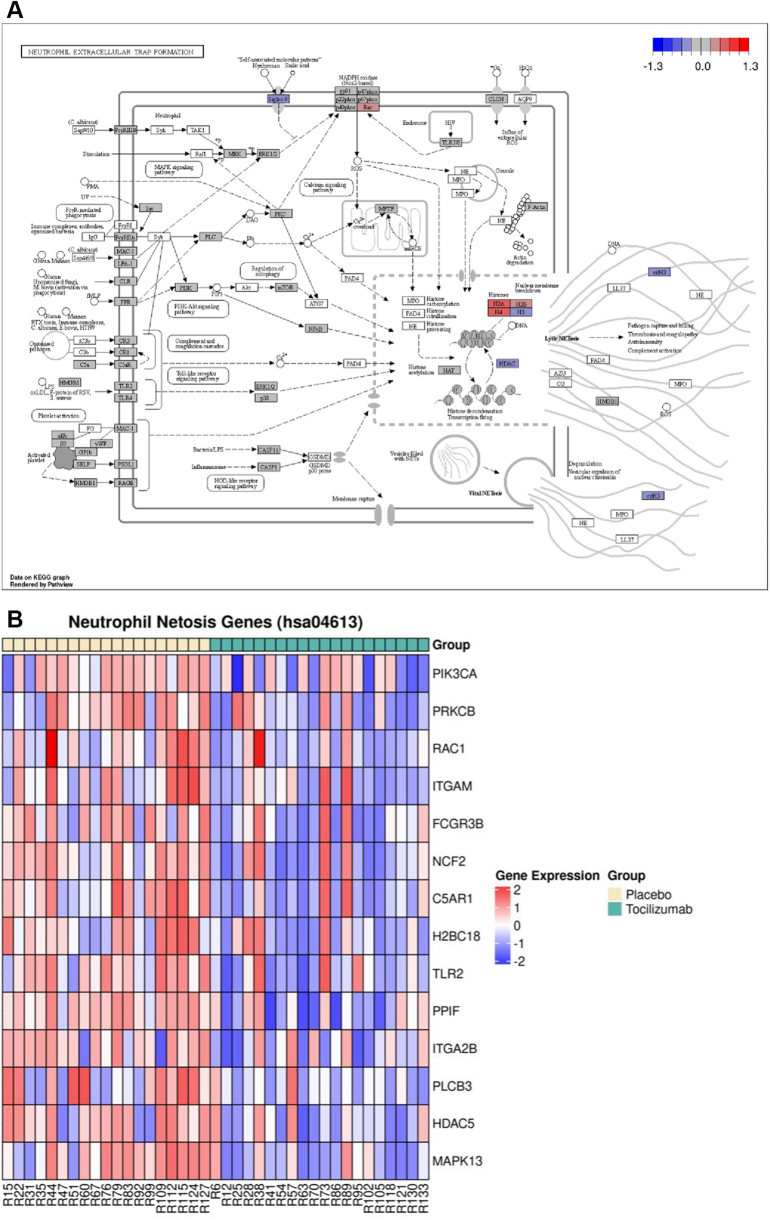
**Transcriptional Regulation of NETosis-Related Pathway by Tocilizumab** (A) Over-representation analysis revealed a significant enrichment of the neutrophil extracellular trap formation pathway (hsa04613) in neutrophil-imputed genes (n = 20). Gray boxes represent enriched genes. Red boxes represent upregulated genes. Blue boxes represent downregulated genes. (B) Tocilizumab decreased regulation of the transcriptional levels of several NETosis-related genes in the extracellular trap formation pathway (hsa04613) compared to placebo (n = 39), *P* < 0.05. *P* values of 2-sample T-test. NET = neutrophil extracellular trap.

Enrichment analysis of neutrophil-imputed genes revealed an overrepresentation of genes belonging to the “Neutrophil Extracellular Trap Formation” pathway at 3 to 7 days (Figure 2A). There was a decrease in the transcriptional levels of histone H3, its citrullinated form, citH3, and histone deacetylases (HDAC), all related to NET formation, in patients treated with tocilizumab. However, there was also a decrease in Siglec-9 expression, suggested to inhibit neutrophil activation. Additionally, there was an increased transcriptional expression of H2A, H2B, and H4, all components of core histones, and, to some extent, Rac (Figure 2A). Of the 89 neutrophil-imputed genes detected in the NETs formation pathway (Supplemental Figure 2), 14 genes were significantly regulated by tocilizumab at an individual gene level, of which the majority were lower in the tocilizumab-treated group (Figure 2B).

### Contribution from NETs on the effect of tocilizumab on myocardial salvage

In ASSAIL-MI, the effect of tocilizumab on the MSI was assessed by linear regression adjusted for time from symptom onset.^3^ We expanded the model with the addition of NET markers separately. As shown in Table 2, lower coefficient values were demonstrated when adding NET markers to the model. The coefficient for the effect of tocilizumab on MSI was 0.06 when only adjusting for time from symptom onset. When adjusting for H3Cit, the coefficient dropped to 0.04. After adjustment for dsDNA, the coefficient dropped further to −0.002.

**Table 2:** The Effect of Tocilizumab on the MSI

Dependent Variable	Independent Variable	Adjusted for	Coefficient	95% CI	*P* Value
MSI	Tocilizumab	Time from symptom onset	0.06	0.002 to 0.113	0.04
		Time from symptom onset H3Cit	0.04	−0.02 to 0.095	0.20
		Time from symptom onset dsDNA	−0.002	−0.064 to 0.059	0.94

dsDNA = double-stranded deoxyribonucleic acid; H3Cit = citrullinated histone 3; MSI = myocardial salvage index.

**Figure 3:**
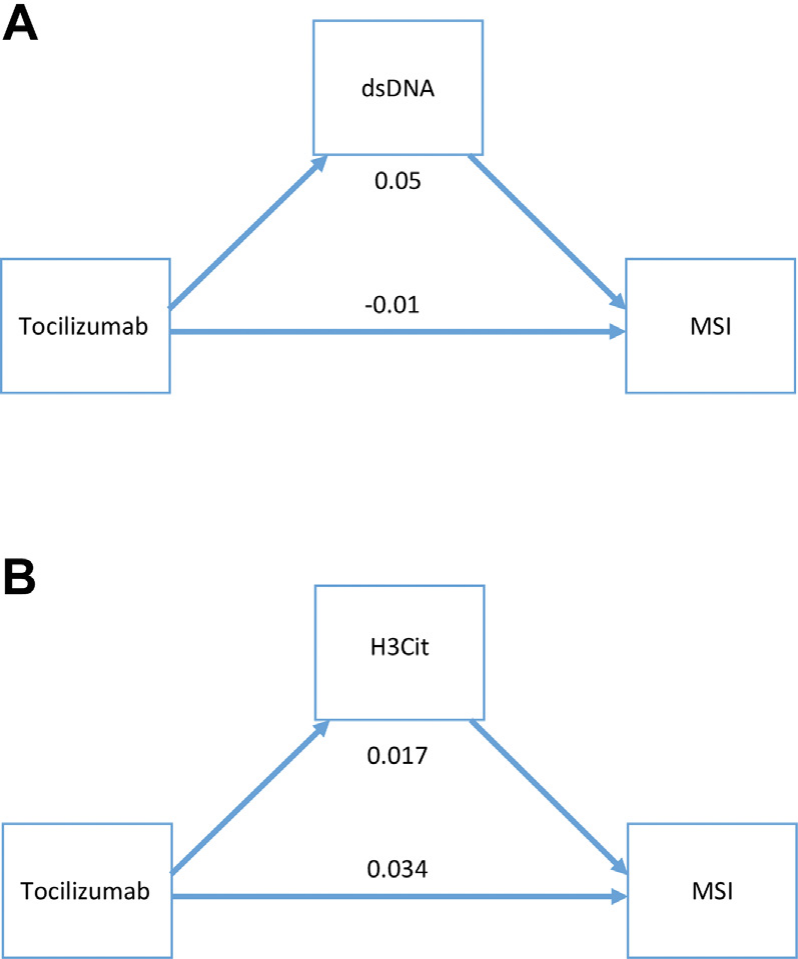
S**tructural Equation Modeling of Effect Pathways of Tocilizumab** Modulation assessment showed path diagram of the direct and indirect effects via dsDNA and H3Cit of tocilizumab on MSI using structural equation modeling. Numbers represent the coefficients of the indirect pathway and direct pathways of NETs markers. (A) dsDNA: indirect pathway (0.05, 95% CI: 0.02 to 0.08, *P* = 0.001) and direct pathway (−0.01, 95% CI: −0.06 to 0.06, *P* = 0.864). (B) H3Cit: indirect pathway (0.017, 95% CI: −0.0003 to 0.336, *P* = 0.055) and direct pathway (0.034, 95% CI: −0.023 to 0.092, *P* = 0.246). dsDNA and H3Cit are measured as continuous variables. dsDNA = double-stranded deoxyribonucleic acid; H3Cit = citrullinated histone 3; MSI = myocardial salvage index.

Structural equation modeling was used to establish the direct and indirect pathways for the effect of tocilizumab on MSI when including H3Cit and dsDNA as mediators. Figure 3 shows how the indirect effect of tocilizumab on the MSI through dsDNA and H3Cit at 3 to 7 days is 0.05 (*P* = 0.001) and 0.02 (*P* = 0.055), respectively, illustrating that attenuated NET levels may contribute to the effect of tocilizumab in ASSAIL-MI.

### NETs and myocardial injury as assessed by cardiac magnetic resonance

**Figure 4:**
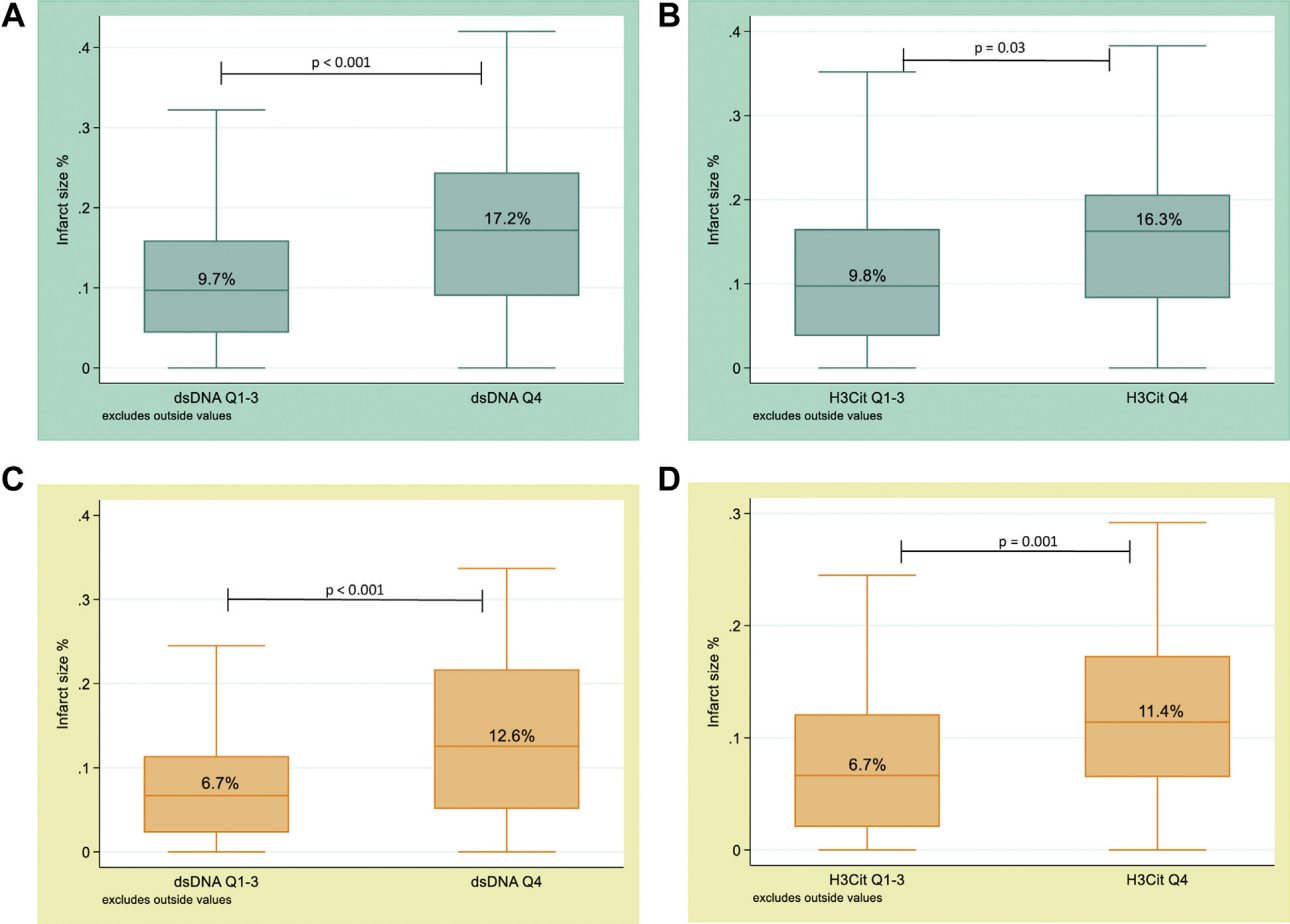
**Association Between Dichotomized NET Markers and Infarct Size** (A and B) Quartile levels of dsDNA (Q1-3: 8-392 ng/ml, Q4: 393-672 ng/ml), H3Cit (Q1-3: 0-3.2 ng/ml, Q4: 3.3-15.5 ng/ml), and infarct size at 3 to 7 days. (C and D) Quartile levels of dsDNA, H3Cit at 3 to 7 days, and infarct size at 6 months. *P* value of Wilcoxon rank-sum test comparing lower 3 quartiles (Q1-3) and Q4. dsDNA = double-stranded deoxyribonucleic acid; H3Cit = citrullinated histone 3; NET = neutrophil extracellular trap.

In a linear regression model adjusted for age, sex, and tocilizumab treatment, dsDNA and H3Cit at 3 to 7 days were significantly associated with the MSI (Table 3). Patients in Q1-3 of dsDNA and H3Cit had larger MSIs (β = 10.9 [95% CI: 3.2-18.6], *P* < 0.006 [dsDNA] and β = 8.9 [95% CI: 2.0-15.9], *P* = 0.01 [H3Cit]). There was no association between MPO-DNA and the MSI. Also, in a linear regression model adjusted for age, sex, and tocilizumab treatment (Table 4), dsDNA and H3Cit levels were both associated with infarct size as assessed by cardiac magnetic resonance imaging after 3 to 7 days and 6 months. Based on the visualization of quartile graphs (Supplemental Figure 3), we dichotomized dsDNA and H3Cit between quartile 3 and 4. Patients in the lowest quartiles of dsDNA (≤392 ng/ml) and H3Cit (≤3.2 ng/ml) had smaller infarct sizes at 3 to 7 days and 6 months than patients in Q4, as shown in Figure 4. Linear regression analyses adjusted for age, sex, and tocilizumab treatment demonstrated the same pattern for the dichotomized NET markers (β = −0.07, 95% CI: −0.01 to −0.032, *P* < 0.001 [dsDNA] and β = −0.05; 95% CI −0.078 to −0-018, *P* = 0.002 [H3Cit] at 3 to 7 days, while β = −0.06, 95% CI: −0.085 to −0.026, *P* < 0.001 [dsDNA] and β = −0.04, 95% CI: −0.067 to −0.014, *P* = 0.003 [H3Cit] after 6 months). Levels of MPO-DNA were not associated with infarct size.

**Table 3:** NET Markers and the MSI

Outcome Variable	Univariable				Multivariable			
	Predictor Variable	Coefficient	95% CI	*P* Value	Coefficient	95% CI	*P* Value	Adjusted R^2^
MSI	dsDNA 3-7 d	−0.097	−0.146 to −0.047	**<0.0001**	−0.092	−0.148 to −0.035	**0.002**^a^	0.069
	H3Cit 3-7 d	−1.84	−3.00 to −0.68	**0.002**	−1.67	−2.85 to −0.49	**0.006**^a^	0.057

All variables are measured as continuous variables. **Bold** indicates P value < 0.05.

dsDNA = double-stranded deoxyribonucleic acid; H3Cit = citrullinated histone 3; MSI = myocardial salvage index; NET = neutrophil extracellular trap.

a Adjusted for age, sex and tocilizumab treatment.

**Table 4:** NET Markers and Infarct Size

Outcome Variable	Univariable				Multivariable			
	Predictor Variable	Coefficient	95% CI	*P* Value	Coefficient	95% CI	*P* Value	Adjusted R^2^
Infarct size% 3-7 d	dsDNA 3-7 d	0.0005	0.0003–0.0007	**<0.001**	0.0005	0.0002–0.0007	**<0.001**^a^	0.11
	H3Cit 3-7 d	0.01	0.005–0.016	**<0.001**	0.01	0.005–0.015	**<0.001**^a^	0.107
Infarct size% 6 mo	dsDNA 3-7 d	0.0005	0.0003–0.0007	**<0.001**	0.0005	0.0003–0.0007	**<0.001**^a^	0.128
	H3Cit 3-7 d	0.008	0.003–0.012	**0.001**	0.01	0.002–0.01	**0.002**^a^	0.083

All variables are measured as continuous variables. **Bold** indicates P value < 0.05.

dsDNA = double-stranded deoxyribonucleic acid; H3Cit = citrullinated histone 3; NET = neutrophil extracellular trap.

a Adjusted for age, sex and tocilizumab treatment. Infarct size as % of left ventricle.

**Figure 5:**
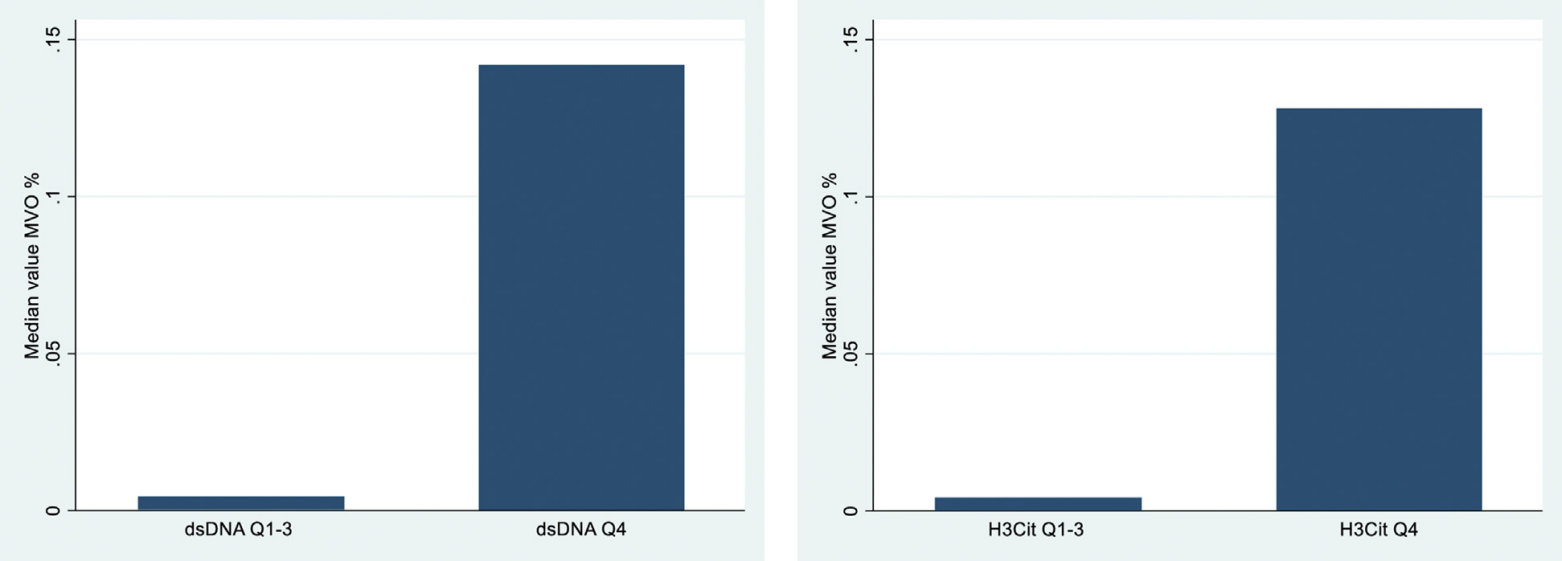
**NET Markers and Microvascular Obstruction** MVO (% of left ventricular volume) in Q1-3 (8-392 ng/ml, n = 139) and Q4 (393-672 ng/ml, n = 46) of dsDNA (left) and Q1-3 (0-3.2 ng/ml, n = 138) and Q4 (3.3-15.5 ng/ml, n = 46) of H3Cit (right) at 3 to 7 days. As the median value of MVO measured as a percentage of the left ventricular volume in the groups with dsDNA Q1-3 and H3Cit Q1-3 were 0, an illustrative bar was added for visual optimization. dsDNA = double-stranded deoxyribonucleic acid; H3Cit = citrullinated histone 3; MVO = microvascular obstruction; NET = neutrophil extracellular trap.

Microvascular obstruction in percent of the left ventricular volume was also significantly lower in Q1-3 vs Q4 for dsDNA (*P* < 0.0001) and H3Cit (*P* = 0.004) (Figure 5). In a logistic regression model adjusted for age, sex, and tocilizumab treatment, Q1-3 of dsDNA and H3Cit were associated with the absence of microvascular obstruction with an OR of 0.42 (95% CI: 0.19-0.92, *P* = 0.03) (dsDNA) and 0.48 (95% CI: 0.24-0.97, *P* = 0.04) (H3Cit).

## Discussion

In this substudy of the ASSAIL-MI trial, we show that tocilizumab reduced NET markers in the acute phase of STEMI (Central Illustration). The ASSAIL-MI trial demonstrated that tocilizumab increased the MSI, and our findings suggest that part of this beneficial effect of tozilizumab could be mediated through reduced formation of NETs. Low levels of the NET markers dsDNA and H3Cit were independently associated with higher MSI, smaller infarct size, and less pronounced microvascular obstruction. The findings highlight the significance of NETs in STEMI and suggest that targeted therapy against IL-6 signaling could attenuate NET formation.

**Central Illustration:**
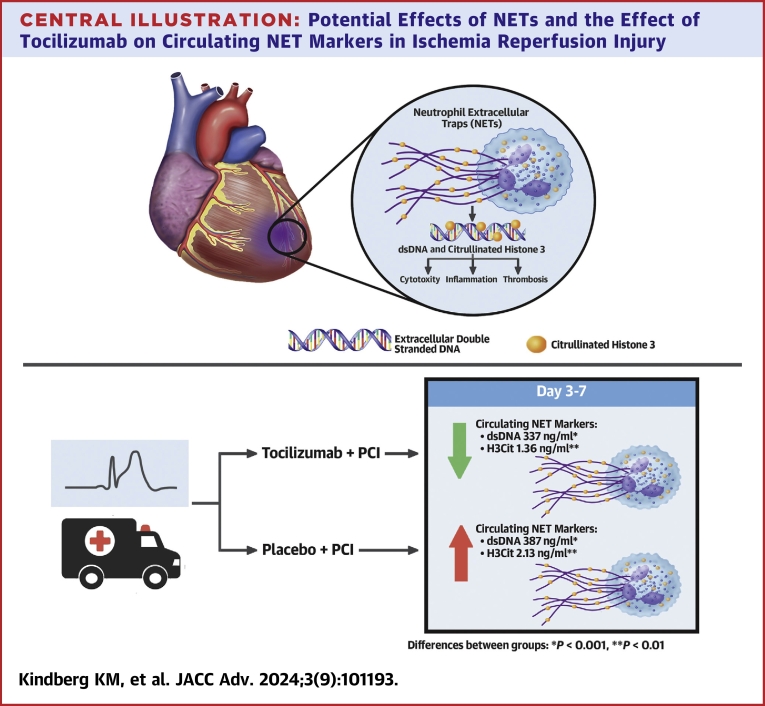
**Potential Effects of NETs and the Effect of Tocilizumab on Circulating NET Markers in Ischemia Reperfusion Injury** NETs contribute to excessive circulating double-stranded DNA (dsDNA) and citrullinated histone 3, which is associated with cytotoxity, inflammation, and thrombosis in ischemia reperfusion after ST-segment elevation myocardial infarction (STEMI). Tocilizumab reduces levels of these circulating NET markers 3 to 7 days after tocilizumab treatment administered prior to percutaneous coronoary intervention (PCI). NET = neutrophil extracellular traps.

Tocilizumab reduced serum levels of all NET markers in this STEMI cohort. Mediation analysis done with structural equation modeling indicated that part of the beneficial effect of tocilizumab on the MSI in the ASSAIL-MI trial could be mediated through NETs. As there may be other mediators of the effect between tocilizumab and MSI that are not included in our model, the results must be reviewed with caution. Nevertheless, our results support a causal association between NET reduction and increased MSI. The reduction at the transcriptional levels of genes related to NET formation suggests that NETs indeed were downregulated by tocilizumab. We have previously shown that the beneficial effect of tocilizumab in ASSAIL-MI might be mediated through reduced numbers of neutrophils and functional attenuation and inhibition of degranulation in these cells.^5^ In the present study, we extend these findings by showing decreased neutrophil transcriptional levels of histone H3, its citrullinated form, citH3, and histone deacetylases, all related to NETs formation. Interestingly, HDAC inhibition has been shown to inhibit NETs formation induced by activated platelets,^20^ and downregulation of HDAC by tocilizumab could be involved in the mechanisms by which tocilizumab attenuate NETs formation in STEMI patients. Moreover, HDAC inhibition has been suggested to attenuate myocardial ischemia reperfusion injury,^21^ further supporting a beneficial role for the tocilicumab-mediated downregulation of HDAC in STEMI. At present, however, the mechanisms by which tocilizumab attenuate NETs formation are not clear. Based on the RNA seq data and the only moderate correlation of NET markers with neutrophil counts, the mechanisms seem to reflect more than just a downregulation of numbers of circulating neutrophils. However, we cannot exclude that the reduced levels of NET markers in the treatment group could involve the restraining effect tocilizumab on the extravascular pool of neutrophil cells, preventing the cells to enter the circulation^22^ and thereby reducing potential NETs release.

Although we find lower levels of NET markers in STEMI patients treated with tocilizumab, we have previously reported increased levels of H3Cit and no changes in dsDNA with tocilizumab treatment in patients with non-ST-elevation myocardial infarction (NSTEMI).^23^ The reason for this discrepancy is not clear but could reflect differences in infarct size, differences in time from symptom onset, and differences in comorbidities and coronary pathophysiology between patients with STEMI and NSTEMI.^24^

Circulating dsDNA and H3Cit were independently associated with the MSI and infarct size in the acute phase and infarct size after 6 months. To our knowledge, this is the first time H3Cit has been associated with MSI and infarct size measured with cardiac magnetic resonance imaging. Although the original trial only found a numerically, not statistically, significant difference in infarct size by treatment groups, one could argue that the statistical analyses were prone to a type II error. We and others have previously shown similar associations between dsDNA and measurements of cardiac function,^7^^,^^15^ and levels of H3Cit have been associated with cardiovascular outcome after STEMI.^25^ There are several proposed mechanisms for how NETs contribute to the myocardial injury in STEMI. Besides enhancing microvascular obstruction,^7^ dsDNA and histones can act as damage-associated molecular patterns,^26^^,^^27^ which through toll-like receptors can lead to immune-mediated cell death.^28^, ^29^, ^30^ Activated toll-like receptors may also activate the nucleotide-binding oligomerization domain-like receptor protein 3 inflammasome in cardiomyocytes,^31^ which further activates the inflammatory cascade of the IL1-IL6 pathway.^17^ Moreover, the pathogenic interactions between platelets, NETs, and endothelial cells are well established in various disorders ^8^^,^^32^ and may be relevant in the setting of STEMI. Also, NETs and H3Cit can stimulate the IL-1-IL-6 pathway,^33^^,^^34^ at least partly through nucleotide-binding oligomerization domain-like receptor protein 3 activation in macrophages, potentially representing a vicious cycle in STEMI. Thus, whereas IL-1 is upstream for IL-6 in this cascade and tocilizumab does not influence IL-1 levels,^35^^,^^36^ the present study shows that targeting IL-6 signaling influences NET formation, potentially contributing to the beneficial effects of tocilizumab in these patients. As tocilizumab inhibits both trans- and classical IL-6 signaling, of which the latter may at least partly have some anti-inflammatory effects,^37^ forthcoming studies could also test the effects of specific inhibition of IL-6 trans-signaling in STEMI patients.

The relationship between serum NET markers and myocardial injury must be interpreted with caution. There are other sources of circulating dsDNA beyond NETs, including necrosis in the area of the myocardial scar. Animal studies have nevertheless repeatedly reported that infarct size can be reduced by reducing NETs.^9^^,^^10^^,^^38^ The association between NETs and myocardial injury support NETs as possible treatment targets in patients with STEMI.

### Study Limitations

This study has several limitations, among which are the modest sample size, the relatively small myocardial infarctions, and the moderate robustness of the laboratory methods. The very modest correlation between the different circulating NET markers illustrates the lack of knowledge on the overall content of NETs in STEMI. Also, the associations between the NET markers and the effect of tocilizumab, as well as the extent of myocardial necrosis, do not necessarily imply causality, although genetic downregulation of genes related to NETs formation along circulating markers were demonstrated. Thrombus NET content has been reported to be associated with clinical outcome in MI,^39^ and although it was not part of the aim of this study, analyses of NETs formation within the intracoronary thrombi would have strengthened the study. Finally, this is a substudy of the primary ASSAIL-MI study and should be interpreted with some caution.

## Conclusions

Treatment with the IL-6 receptor inhibitor tocilizumab reduced NETs in patients with STEMI. The beneficial effect of tocilizumab on the MSI might be mediated through reduction of NETs. Low serum NET levels were independently associated with increased MSI, smaller infarct size, and less microvascular obstruction. Overall, these results support an important role for NETs in STEMI and that IL-6 inhibition attenuates NETs.

Perspectives **COMPETENCY IN MEDICAL KNOWLEDGE:** In a substudy of the ASSAIL-MI trial, we show how a single dose of the interleukin-6 inhibitor tocilizumab in patients with STEMI reduces NETs and how NETs associate with myocardial injury.

**TRANSLATIONAL OUTLOOK:** Trials designed to address 1) effects of NET reduction in STEMI and 2) the role of NETs in myocardial ischemia reperfusion injury are necessary.

## Funding support and author disclosures

This work was supported by the 10.13039/501100006095South-Eastern Norway Regional Health Authority. Roche provided the investigational medicinal products and an unrestricted grant for the ASSAIL-MI study. Dr Broch has received lecture and consultant fees from Amgen, AstraZeneca, Bohringer Ingelheim, Merck, MSD, Novartis, Novo Nordisk, Pfizer, Pharmacosmos, and Vifor Pharma. Dr Gullestad has received lecture fees from AstraZeneca, Boehringer Ingelheim, Novartis, and Amgen; and has been a member of local advisory board in AstraZeneca and Boehringer Ingelheim. All other authors have reported that they have no relationships relevant to the contents of this paper to disclose.
