# Supplementary material for: Neutrophil Extracellular Traps in ST-Segment Elevation Myocardial Infarction: Reduced by Tocilizumab and Associated With Infarct Size
Source: JACC Adv. 2024 Aug 15;3(9):101193. doi: 10.1016/j.jacadv.2024.101193 (PMC11378880; doi:10.1016/j.jacadv.2024.101193)
Supplement: Supplementary material [file mmc1.docx]

**Supplemental table 1.** Technical specifications on MPO-DNA and H3Cit Enzyme-linked immunosorbent assay.

|  | Capture antibody | LOT number |
| --- | --- | --- |
| MPO-DNA complex | Anti-MPO MAb (ABD Serotec, Bio-Rad Norway, Cat-No. 0400-0002) | 61497200 (all kits) |
| H3Cit | Monoclonal antibody(MAb) specific for histone H3 (citrullinated at R2, R8, and R17). | 0645225 (11 of 12 kits), 0652059 (1 of 12 kits) |

**Supplemental table 2**. NET markers correlation with both neutrophil cells and platelets.

|  | Neutrophil cells | Spearman’s rho | p-value | Platelets | Spearman’s rho | p-value |
| --- | --- | --- | --- | --- | --- | --- |
| dsDNA at 24 hours | **Neutrophils at 24 hours** | **0.211** | **0.004** | **Platelets at 24 hours** | 0.044 | 0.56 |
| MPO-DNA at 24 hours |  | **0.266** | **0.0002** |  | 0.075 | 0.31 |
| H3cit at 24 hours |  | 0.016 | 0.83 |  | 0.054 | 0.47 |
| dsDNA at 3-7 days | **Neutrophils at 3-7 days** | **0.340** | **<0.0001** | **Platelets at 3-7 days** | -0.033 | 0.66 |
| MPO-DNA at 3-7 days |  | 0.135 | 0.068 |  | 0.068 | 0.36 |
| H3cit at 3-7 days |  | **0.232** | **0.002** |  | -0.079 | 0.29 |
| ∆dsDNA BL to 3-7 days | **∆Neutrophils from BL to 3-7 days** | **0.193** | **0.011** | **∆Platelets from BL to 3-7 days** | **-0.152** | **0.04** |
| ∆MPO-DNA BL to 3-7 days |  | **0.247** | **0.001** |  | -0.004 | 0.96 |
| ∆H3cit BL to 3-7 days |  | **0.181** | **0.018** |  | 0.016 | 0.83 |

**Supplemental figure 1:** Association between dichotomized NET markers and infarct size included
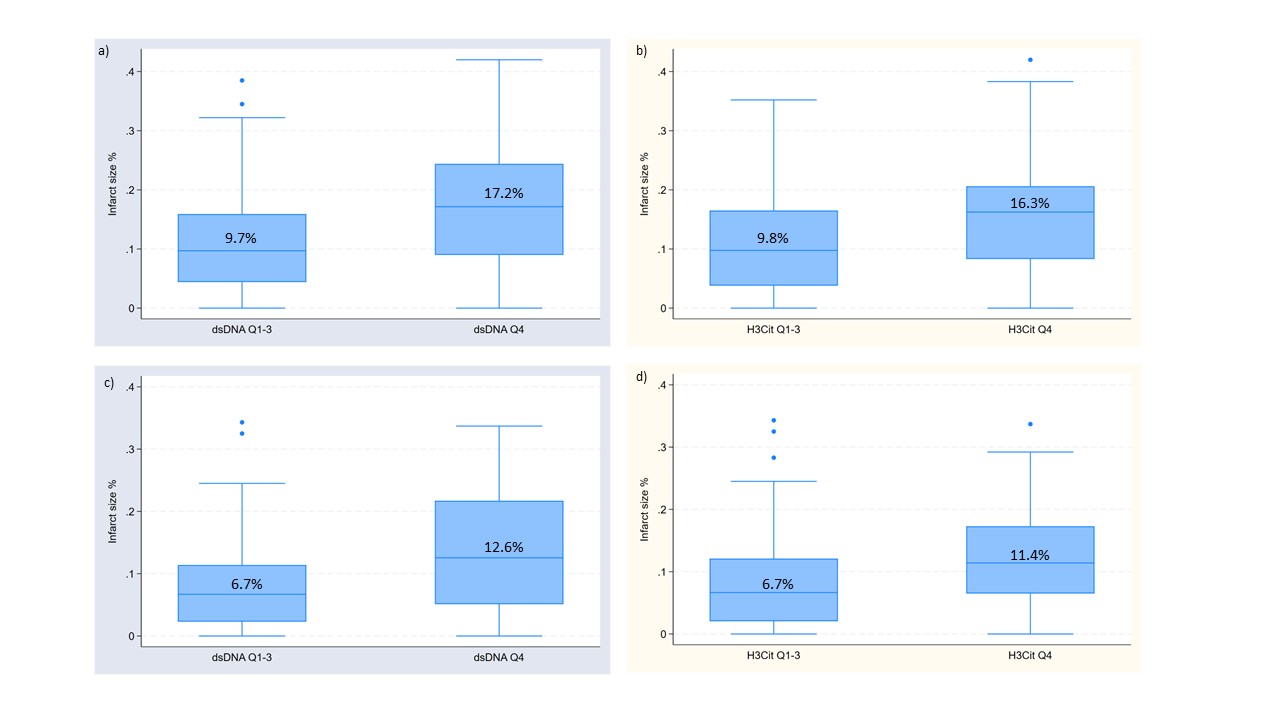
outliers.

**Supplemental figure 1.** a) and b): Quartile levels of dsDNA (Q1-3: 8-392 ng/ml, Q4: 393-672 ng/ml), H3Cit (Q1-3: 0-3.2 ng/ml Q4: 3.3-15.5 ng/ml) and infarct size at 3-7 days. c) and d): Quartile levels of dsDNA, H3Cit at day 3-7 and infarct size at 6 months. dsDNA: double stranded deoxyribonucleic acid. H3Cit: citrullinated Histone 3.

**Supplemental figure 2:** Neutrophil Extracellular Trap formation genes detected in neutrophil-imputed genes.


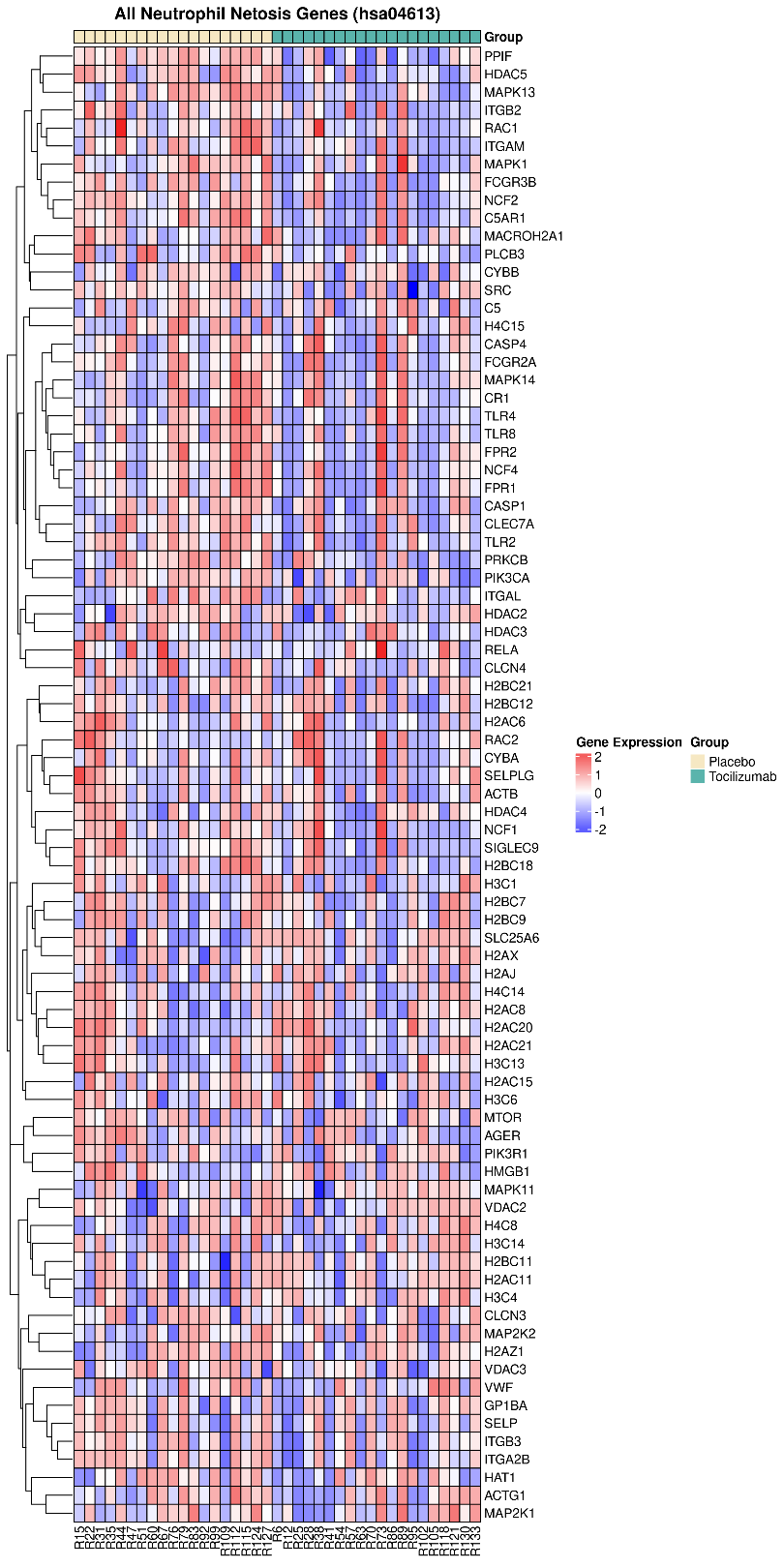


**Supplemental figure 2.** 89 neutrophil-imputed genes were recognised as part of the Neutrophil Extracellular Trap formation pathway. The relative expression of neutrophil-imputed genes reveals a tendency for the overall lowered expression of neutrophilic extracellular trap formation genes in the tocilizumab group (n=20) compared to the control group (n=19).

**Supplemental figure 3:** Median value of the quartiles of dsDNA and H3Cit at 3-7 days.

**Supplemental figure 3:** Presentation of the median values of the quartiles of dsDNA (a) and H3Cit (b). The number represent the median value of the quartile. The red line represent the cut off between Q1-3 and Q4.

b)

))

a)
